# Phytochemical composition of wormwood (*Artemisia gmelinii*) extracts in respect of their antimicrobial activity

**DOI:** 10.1186/s12906-019-2719-x

**Published:** 2019-10-28

**Authors:** Aliya S. Mamatova, Izabela Korona-Glowniak, Krystyna Skalicka-Woźniak, Aleksandra Józefczyk, Krzysztof K. Wojtanowski, Tomasz Baj, Zuriyadda B. Sakipova, Anna Malm

**Affiliations:** 1 JSC Asfendiyarov Kazakh National Medical University, Republic of Kazakhstan, 94 Tolebi Str, Almaty, 050000 Republic of Kazakhstan; 2 Department of Pharmaceutical Microbiology with Laboratory for Microbiological Diagnostics of Medical University of Lublin, 1 Chodzki Street, 20-093 Lublin, Poland; 3 Department of Pharmacognosy with Medicinal Plant Unit of Medical University of Lublin, 1 Chodzki Street, 20-093 Lublin, Poland

**Keywords:** *Artemisia gmelinii*, Asteraceae, Phytochemical analysis, Antimicrobial activity

## Abstract

**Background:**

Extracts from medicinal plants with phytochemicals with known antimicrobial properties can be an effective adjunct in the complex treatment of infectious diseases. This study aimed to evaluate the antimicrobial activity of wormwood extracts collected in Kazakhstan (*Artemisia gmelinii* Weber ex Stechm.), along with their phytochemical analysis.

**Methods:**

The ethanolic and chloroform extracts were subjected to HPLC combined with quadrupole time-of-flight mass spectrometry method. For quantitative assessment of antimicrobial activity, minimal inhibitory concentration (MIC) of the tested extracts was determined by micro-dilution broth method for the panel of the reference microorganisms. Minimal bactericidal concentration (MBC) or minimal fungicidal concentration (MFC) were also determined.

**Results:**

LC/MS analysis showed the presence of 13 compounds in the tested extracts, including flavonoids: apigenin, luteolin, rutin, two O-methylated flavonols (isorhamnetin, rhamnazine), coumarin compounds (umbelliferone, scopoletin and scopolin (scopoletin 7-glucoside), 3-hydroxycoumarin and 4-hydroxycoumarin), chlorogenic acid and two dicaffeoylquinic acid isomers. Quantitative HPLC analysis showed that umbelliferone was dominant in the chloroform extract while chlorogenic acid was identified as a main compound in the ethanolic extract. The antibacterial and antifungal activity of chloroform and ethanolic extracts was comparable. The most sensitive were the Gram-positive bacteria represented by staphylococci, *Micrococcus luteus* and *Bacillus* spp. (MIC = 1.25–5 mg/ml) and yeasts represented by *Candida* spp. (MIC = 2.5–5 mg/ml), irrespective of the assayed extract.

**Conclusions:**

Extracts of wormwood *Artemisia gmelinii* have shown a wide spectrum of antibacterial and antifungal activity. Luteolin, rutin, isorhamnetin and scopolin were identified in *A. gmelinii* species for the first time. The determining of the most potential compounds of *Artemisia gmelinii* can be used to develop effective antibacterial and antifungal agents.

## Background

*Artemisia gmelinii* Webb & Stechmann, also known as Gmelin’s wormwood, is one from over 500 species in the genus *Artemisia* (*Asteraceae*). It is a perennial plant, reaching up to 50–150 cm of height, richly-branched, grayish-brown color, strongly woody at the bottom. Taproot with a thickness of 3–5 cm. Leaves are thick and mossy especially on the underside, however loosely on the upper side. Inflorescences are spherical (20–30 in head), small, slightly drooping, densely arranged, gathered in a short panicles. The fruit is achene (1,5 mm) finely – striped, brown. They bloom from August to October [1, 2]. The plant occurs on the dry stony and rocky slopes, hills, semi-deserts, grasslands, wastelands, at the altitude of 1500–4900 m asl from Eastern Europe to Central Asia, China, Mongolia and Korea. Brought to the area of the United States, growing endemically in the state of Ohio. The plant’s preference is a sandy and loamy, well drained soil [2].

The leaf and stem are used in Korea to treat hepatitis, hyperlipaemia and infected cholecystitis with flavonoids and sesquiterpenes being suggested as bioactive constituents. Dried parts of the plant have been applied in Tradition Chinese Medicine for treatment and prevention of chronic inflammations of the liver [3]. As the mechanism of action the inhibitive effects of lipid peroxidation and the down-regulation of TNF-α mediated apoptosis were suggested [3].

Biologically active substances (BAS) derived from medicinal plants can be an effective adjunct in the complex treatment of infectious diseases [4–7]. Different *Artemisia* species are described as producers of metabolites with antimicrobial and antioxidant activity. They were reported for high content of alkaloids, flawonoids, phenol, quinines and terpenoids [8–10]. Phytochemical studies concerning phytochemical composition of *A. gmelini* are rather partial. So far 1,8 - cineol (21–40%), camphora (10–31%), borneol (4–17%) were detected as main compounds in essential oil [11], as well as polyphenolic components such as scopoletin, chlorogenic acid, 4′,7- di-O-methyl-apigenin, acacetin and 4′,5,7 - trihydroxy-3′,6-dimethoxyflavon [12], caffeic acid, 4-O-caffeoylquinic acid, luteolin-7-O-glucoside, and apigenin-7-O-glucoside, 3,5-O-dicaffeoylquinic acid and its ethyl ester derivatives [13] were indicated as dominant compounds of *Artemisia gmelinii*.

As *A. gmelinni* is distributed widespread in the area of Kazakhstan and both composition and application are not well defined, the aim of the present studies was the evaluation of the antimicrobial activities of extracts of wormwood collected in Kazakhstan (*Artemisia gmelinii* Weber ex Stechm.) along with their preliminary phytochemical analysis.

## Methods

### Plant material and extraction

The aerial parts of wormwood were collected in July and August 2015 in the generative phase at the foothills of the Trans-Ili Alatau of the Republic of Kazakhstan. Raw material drying was carried out by air-shadow method, in the well-ventilated accommodations, out of direct sunlight. The harvesting was produced in accordance with the principles of the GACP standard. The collected raw materials were identified in accordance with the requirements of the State Pharmacopoeia of the Republic of Kazakhstan by Danylov Mikhail Petrovich, botanic specialist in Botanical Garden, Institute of Botany and Phytointroduction of Kazakhstan Academy of Sciences in Almaty, Kazakhstan. The vouchers have been deposited in herbarium of Institute of Botany and Phytointroduction of Kazakhstan Academy of Sciences, receiving identification numbers ASM-AG 2015/01.

A technology for obtaining dry extract from the raw material of wormwood with the use of 70% ethanol by percolation has been developed. Extraction of BAS was due to convective diffusion to complete depletion, then further purification and thickening of the extract to a moisture content of no more than 5% (dry extract) was carried out. The method of obtaining 100 g of dry extract from 4 kg of raw materials consisted of the following steps: soaking of raw materials, maceration pause (infusion), percolation (direct transition of the extractant through the raw material layer), purification and thickening of the extract as well as identification of the extract. The extract output was 2.5% ± 0.26.

The preparations of 30 g of thick extract from 1455 g of raw material by the method of circulation extraction with the use of a volatile chloroform solvent were carried out in a classical manner in a “Soxhlet” type installation. The resulting thick extract was a viscous mass of dark brown color, with a specific smell of a bitter taste. The extract output was 2.1% ± 0.71.

Exactly 20 mg of each extracts were weighed and then dissolved in 10 ml of a mixture of methanol-water 8:2 v/v. Extracts were subjected to fingerprint analysis with application of HPLC after passing syringe filters (PTFE, 0.45 μm).

### Reagents

Standard substances for HPLC analysis were purchased in the Sigma-Aldrich® Co. and ChromaDex Inc.

### HPLC analysis

Extracts were analyzed using an Agilent Technologies 1100 liquid chromatograph with a visible diode – array detector (DAD) equipped with Zorbax Eclipse XDB C8 column (150 X 4.6 mm I.D., dp = 5 μm) with gradient elution: A- water + 1% acetic acid; B – acetonitryle (0 min- 10% B; 0–10 min - 10-14% B; 10–25 min - 14-30% B; 25–35 min. – 30-35% B; 35–50 min. - 35-60% B; 50–57 min. 60% B). Injection volume for extracts and standards was 10 μL. The flow rate was 1 ml/min and column thermostat temp. 25 °C. The identification of the compounds was performed by comparing retention times and UV-DAD spectra (λ = 254, 280 and 325 nm) with those for standard solutions. Quantitative determination was performed on the basis of 3-fold determinations of the tested compounds. Determination of the content of the tested compounds in individual extracts was determined with the use of calibration curves and calculated mathematically. Calibration plot was prepared of five different concentrations (1, 0.75, 0.5, 0.25, 0.1 mg per 10 mL for all investigated coumarins, phenolic acids and flavonoids. In the HPLC-DAD analysis of compounds of examined extract calibration plots were highly linear – R^2^ > 0,9932 for phenolic acids R^2^ > 0,9966 for flavonoids and R^2^ > 0,9867 for coumarins.

### LC-MS analysis

The purified samples were analysed quantitatively by an HPLC/ESI-QTOF-MS system in positive ion mode using a 6530B Accurate-mass-QTOF-MS (Agilent Technologies, Inc., Santa Clara, CA, USA) mass spectrometer with an ESI-Jet Stream ion source. The chromatograph was equipped with DAD detector, autosampler, binary gradient pump, and column oven. Gradient of solvents: acetonitrile (1%) with 10 mM ammonium formate (0.1%) (solvent A) and acetonitrile (95%) with 10 mM ammonium formate (0.1%) (solvent B) were used as the mobile phases. The following gradient procedure was adopted: 0–10 min, 10–14% of (B); 10–15 min, 14–22% (B); 15–25 min, 22–30% (B), 25–35 min, 30–35% (B),35–45 min 35–55% (B), 45-47 min, 55–65% (B) and 47–48, 65–80% (B). Total time of analysis was 48 min, with a stable flow rate at 0.200 mL/min. Injection volume for extracts was 10 μL. ESI-QTOF-MS analysis was performed according to the following parameters of the ion Source: Dual spray jet stream ESI, positive and negative ion mode, gas (N2) flow rate: 12 L/min., nebulizer pressure: 35 psig, vaporizer temp.: 300 °C; m/z range 100–1000 mass units, with acquisition Mode AutoMS/MS, collision induced dissociation (CID): 200 eV with MS scan rate 1 spectrum per s, 2 spectra per cycle, skimmer: 65 V, fragmentor: 150 V and octopole RF Peak: 750 V. Qualitative and quantitative analysis of the extract from TF was made additionally in auto MS/MS with excluded: m/z at 922.0097 and 121.0508 for positive ion mode and 966.0007 and 112.9855 for negative ion mode corresponding to the m/z of reference ions.

Identification of compounds was based on Metlin database (https://metlin.scripps.edu) and compared with literature data.

### GC-MS analysis

GC-MS was performed with a Shimadzu GC-2010 Plus gas chromatography instrument coupled to a Shimadzu QP2010 Ultra mass spectrometer equipped with fused silica capillary column ZB-5 MS (30 m, 0.25 mm i.d.) with a film thickness of 0.25 μm (Phenomenex). Analysis were performed according to previously published method [14]. The oven temperature program was initiated at 50 °C, held for 3 min, then increased at the rate of 8 °C min-1 to 250 °C, held for 2 min. The spectrometers were operated in electron-impact (EI) mode, the scan range was 40–500 amu, the ionization energy was 70 eV and the scan rate was 0.20 s per scan. Injector, interface and ion source were kept at 250, 250 and 220 °C, respectively. Split injection (1 μL) was conducted with a split ratio of 1:20 and helium was used as carrier gas of 1.0 mL min-1 flow-rate. The retention indices were determined in relation to a homologous series of n-alkanes (C8-C24) under the same operating conditions. Thujone was identified using a computer-supported spectral library [15].

### Antimicrobial activity assay in vitro

The ethanolic and chloroform extracts were screened for antibacterial and antifungal activities by micro-dilution broth method according to both the European Committee on Antimicrobial Susceptibility Testing (EUCAST) (www.eucast.org) using Mueller-Hinton broth and Mueller-Hinton broth with 5% lysed sheep blood for growth of non-fastidious and fastidious bacteria, respectively or RPMI with MOPS for growth of fungi as we described elsewhere [16]. Minimal Inhibitory Concentration (MIC) of the tested derivatives were evaluated for the wide panel of the reference microorganisms from American Type Culture Collection (ATCC), including Gram-negative bacteria (*Escherichia coli* ATCC 25922, *Salmonella* Typhimurium ATCC14028, *Klebsiella pneumoniae* ATCC 13883, *Pseudomonas aeruginosa* ATCC 9027, *Proteus mirabilis* ATCC 12453), Gram-positive bacteria (*Staphylococcus aureus* ATCC 25923, *Staphylococcus aureus* ATCC 6538, *Staphylococcus epidermidis* ATCC 12228, *Micrococcus luteus* ATCC 10240, *Bacillus subtilis* ATCC 6633, *Bacillus cereus* ATCC 10876*, Streptococcus pyogenes* ATCC 19615, *Streptococcus pneumoniae* ATCC 49619, *Streptococcus mutans* ATCC 25175) and fungi (*Candida albicans* ATCC 10231, *Candida parapsilosis* ATCC 22019). Each experiment was repeated in triplicate. Representative data is presented.

### Statistical analysis

All analysis were done in triplicates in order to prove their reproducibility. The results were expressed as mean ± SD. Mann-Whitney test using GraphPad InStat 3 was performed to determine the significance of MIC values between the extracts. Statistical significance was set at *p* < 0.05.

## Results

### Phytochemical analysis

Prior to comprehensive sample analysis, extracts from *A. gmelinii* with different polarities (ethanol, chloroform) were subjected to HPLC-DAD and LC-MS analysis in order to systemise the knowledge about phytochemical composition. Preliminary HPLC analysis resulted in identification of: umbelliferone (1), chlorogenic acid (2), rutin (3), scopoletin (7), luteolin (10) and apigenin (12). The numbering in parentheses is assigned according to the results of the following LC-MS analysis. Other unknown compounds (coumarins, flavonoids and phenolic acid derivatives) were also detected. Obtained HPLC–DAD (λ = 325 nm) chromatograms are presented on Additional files 1 and 2: Figures S1, S2. Quantitative determination of the compounds identified by this method was performed on the basis of 3-n determinations and converted into the content in 1 g of investigated extracts [mg/g]. The content of identified compounds is presented in Table 1. In both investigated extracts umbelliferone was identified, with 3-times higher content in chloroform extract compared to ethanolic. A simple coumarin scopoletin as well as two flavones - luteolin, apigenin were detected in chloroform extract and absent in ethanolic one. Chlorogenic acid – a polar depside, was dominant in ethanolic extract and absent in chloroform one. Additionally rutin was detected.

**Table 1:** HPLC- DAD analysis. The content of identified compounds in the extracts from *A. gmelinii* Weber ex Stechm. per gram of plant material used [mg/g] and (SD; RSD)

Name	No	chloroform extract	ethanolic extract
Umbelliferone	1	10.96 (0.0011; 0.103)	3.46 (0.005; 0.074)
Chlorogenic acid	2	nd	156.98 (0.032; 0.18)
Rutin	3	nd	7,03 (0.021; 0.144)
Scopoletin	7	10.05 (0.031; 0.176)	nd
Luteolin	10	5.07 (0.028; 0,167)	nd
Apigenin	12	1.11 (0.0002; 0.014)	nd

To confirm this, as well as identified other active constituents, ethanolic and chloroform extracts were subjected to LC-MS. 13 compounds were identified, as described below and listed in Table 2. Obtained chromatograms are presented on Additional files 3, 4 and 5: Figures S3-S5.

**Table 2:** Compounds identified by LC-MS in ethanolic and chloroform extracts from *A. gmeilinii*

	Name / extract	[M-H]^−^	[M + H]^+^	[M + Na]^+^	ms/ms	Extract
1	Umbelliferon	161.0229			133.0313	Ethanolic
			163.0371		107.0494; 91.0550	
2	Chlorogenic acid	353.0889			191.0562	Ethanolic
3	Rutin	609.1494			300.2078; 271.0242; 255.0310; 151.0020	
				633.1440	331.1003; 324.0205	
4,5	Di-caffeoylquinic acid isomers	515.1207			353.0893; 191.0561; 179.0350; 161.0239	Ethanolic
				539.1178	377.0828; 359.0753; 215.0516; 185.0187	
6	Scopolin			377.0817	215.0304	Ethanolic
7	Scopoletin		193.0491		178.0268; 161.0220; 150.0305; 137. 0596; 133.0280; 122.0361; 105.0280	Ethanolic
8	4-hydroxycoumarin	161.0249			133.0297; 117.0333	Chloroform
9	3-hydroxycoumarin	161.0241			–	Chloroform
10	Luteolin	285.0406			175.0397; 151.0023; 133.0282	Chloroform
11	Isorhamnetin	315.0513			300.0276; 285.0405; 271.0248; 255.0299; 151.0039	Chloroform
12	Apigenin	269.0450			225.0554; 151.0018; 149.0244; 117.0329	Chloroform
13	Rhamnazin	329.0679			314.0450; 299.0209; 151.0031	Chloroform

The ESI-TOF-MS/MS spectrum of **compound 1** in the negative ion mode at exhibited the molecular ion peak [C_9_H_5_O_3_]^−^ at m/z = 161.0229 (theoretical mass 161.0244; − 9.37 ppm error). The compound was identified as **umbelliferon**. The ESI-TOF-MS/MS spectrum of **compound 2** in the negative ion mode exhibited the molecular ion peak [C_16_H_17_O_9_]^−^ at m/z = 353.0889 (theoretical mass 353.0878; − 3.09 ppm error). The compound was identified as **chlorogenic acid** or its isomer. The ESI-TOF-MS/MS spectrum of **compound 3** in the negative ion mode at exhibited the molecular ion peak [C_27_H_29_O_16_]^−^ at m/z 609.1494 (theoretical mass 609.1450; − 5.39 ppm error). Furthermore, the ESI-TOF-MS/MS spectrum of **compound 3** in the positive ion mode at exhibited the sodium adduct of molecular ion peak [C_27_H_30_O_16_Na]^+^ at m/z = 633.1440 (theoretical mass 633.1426; − 2.21 ppm error) The compound was identified as **rutin**. The ESI-TOF-MS/MS spectrum of **compound 4** in the negative ion mode at exhibited the molecular ion peak [C_25_H_23_O_12_]^−^ at m/z 515.1207 (theoretical mass 515.1195; − 2.33 ppm error), additionally the ESI-TOF-MS/MS spectrum of **compound 4** in the positive ion mode at exhibited the sodium adduct of molecular ion peak [C_25_H_24_O_12_Na]^+^ at m/z 539.1178 (theoretical mass 539.1160; − 3.35 ppm error) The compound was identified as **dicaffeoylquinic acid isomer**. The ESI-TOF-MS/MS spectrum of **compound 5** in the negative ion mode at exhibited the molecular ion peak [C_25_H_23_O_12_]^−^ at m/z 515.1209 (theoretical mass 515.1195; − 2.71 ppm error) also the ESI-TOF-MS/MS spectrum of **compound 5** in the positive ion mode at exhibited the sodium adduct of molecular ion peak [C_25_H_24_O_12_Na]^+^ at m/z 539.1164 (theoretical mass 539.1160; − 0.75 ppm error) The compound was identified as **dicaffeoylquinic acid isomer**. The ESI-TOF-MS/MS spectrum of **compound 6** in the positive ion mode at exhibited the molecular ion peak [C_16_H_18_O_9_Na]^+^ at m/z 377.0817 (theoretical mass 377.0843; 6.92 ppm error) The compound was identified as **scopolin**. The ESI-TOF-MS/MS spectrum of **compound 7** in the positive ion mode at exhibited the molecular ion peak [C_10_H_11_O_4_]^+^ at m/z 193.0491 (theoretical mass 193.0495; 2.27 ppm error) The compound was identified as **scopoletin**. The ESI-TOF-MS/MS spectrum of **compound 8** in the negative ion mode at exhibited the molecular ion peak [C_9_H_5_O_3_]^−^ at m/z 161.0491 (theoretical mass 161.0249; − 2.98 ppm error) The compound was identified as **4-hydroxycoumarin**. The ESI-TOF-MS spectrum of **compound 9** in the negative ion mode at exhibited the molecular ion peak [C_9_H_5_O_3_]^−^ at m/z = 161.0241 (theoretical mass 161.0249; 1.96 ppm error). The compound was identified as **3-hydroxycoumarin**. The ESI-TOF-MS/MS spectrum of **compound 10** in the negative ion mode at exhibited the molecular ion peak [C_15_H_9_O_6_]^−^ at m/z 285.0406 (theoretical mass 285.0405; − 0.48 ppm error) The compound was identified as **luteolin**. The ESI-TOF-MS/MS spectrum of **compound 11** in the negative ion mode at exhibited the molecular ion peak [C_16_H_11_O_7_]^−^ at m/z = 315.0513 (theoretical mass 315.0510; − 0.87 ppm error) The compound was identified as **isorhamnetin**. The ESI-TOF-MS/MS spectrum of **compound 12** in the negative ion mode at exhibited the molecular ion peak [C_15_H_9_O_5_]^−^ at m/z = 269.0450 (theoretical mass 269.0455; 2.03 ppm error). The compound was identified as **apigenin**. The ESI-TOF-MS/MS spectrum of **compound 13** in the negative ion mode at exhibited the molecular ion peak [C_17_H_13_O_7_]^−^ at m/z = 329.0679 (theoretical mass 329.0667; − 3.71 ppm error) The compound was identified as **rhamnazin**.

Quantitative determination of known toxic phytochemicals thujone was performed by GC-MS method for both extracts. The content was between 0,12-0,16% - thus we assume as low and without influence on the activity.

### Antimicrobial activity

Reference strains of bacteria and fungi enlisted in Table 3 were taken for preliminary screening of antimicrobial activity using micro-broth dilution method. It was shown that the assayed extracts inhibited growth of the reference microorganisms with MIC of 1.25 - > 20 mg/ml, depending on the strains and solvents used. The biological activity of chloroform and ethanolic extracts was comparable (Mann-Whitney test, *p* = 0.78). The most sensitive were gram-positive bacteria represented by staphylococci, *Micrococcus luteus* and *Bacillus* spp. (MIC = 1.25–5 mg/ml) and yeasts represented by *Candida* spp. (MIC = 2.5–5 mg/ml), irrespective of the assayed extract. The lowest biological activity of both extracts was observed against gram-negative rods

**Table 3:** Antimicrobial activity of ethanolic and chloroform *Artemisia gmelinii* extracts and reference compounds – vancomycin, ciprofloxacin for bacteria and fluconazole for yeasts

Plant extracts	chloroform extract			ethanolic extract			Vancomycin		
Bacteria	MIC (mg/mL)	MBC (mg/mL)	MBC/MIC ratio	MIC (mg/mL)	MBC (mg/mL)	MBC/MIC ratio	MIC-(μg/mL)	MBC-(μg/mL)	MBC/MIC ratio
Gram-positive bacteria									
*Staphylococcus aureus* ATCC 25923	5	20	4	2.5	2.5	1	0.98	7.81	8
*Staphylococcus aureus* ATCC 6538	5	20	4	5	5	1	0.49	1.95	4
*Staphylococcus aureus* ATCC 43300	1.25	5	>4	1.25	5	4	0.49	1.95	4
*Staphylococcus epidermidis* ATCC 12228	5	>20	>4	2.5	2.5	1	0.98	0.95	1
*Micrococcus luteus* ATCC 10240	5	>20	>4	5	10	2	0.12	0.12	1
*Bacillus subtilis* ATCC 6633	5	10	2	5	20	4	0.24	0.49	2
*Bacillus cereus* ATCC 10876	5	>20	>4	5	>20	>4	0.98	16.6	16
*Streptococcus pyogenes* ATCC 19615	5	5	1	5	5	2	0.24	0.49	2
*Streptococcus pneumoniae* ATCC 49619	2.5	2.5	1	5	5	2	0.24	0.49	2
*Streptococcus mutans* ATCC 25175	10	10	1	10	10	1	0.98	0.98	1
Gram-negative bacteria							Ciprofloxacin		
*Escherichia coli* ATCC 25922	> 20	> 20	nd	20	20	1	0.004	0.004	1
*Proteus mirabilis* ATCC 12453	> 20	> 20	nd	10	10	1	0.03	0.03	1
*Klebsiella pneumoniae* ATCC 13883	20	20	1	5	5	1	0.12	0.12	1
*Pseudomonas aeruginosa* ATCC 9027	> 20	> 20	nd	20	20	1	0.49	0.98	2
Yeasts	MIC (mg/ml)	MFC (mg/ml)	MFC/MIC ratio	MIC (mg/ml)	MFC (mg/ml)	MFC/MIC ratio	Fluconazole		
							MIC (μg/mL)	MFC (μg/mL)	MFC/MIC ratio
*Candida albicans* ATCC 102231	2.5	5	2	5	10	2	0.98	1.95	2
*Candida albicans* ATCC 2091	5	5	1	5	10	2	0.25	0.25	1
*Candida parapsilosis* ATCC 22019	5	10	2	2.5	10	4	1.95	1.95	1

*MIC* minimal inhibitory concentration, *MBC* minimal bactericidal concentration, *MFC* minimal fingicidal concentration

## Discussion

Different *Artemisia* species are described as producers of metabolites with an antimicrobial activity [10, 17–19]. However, to our best knowledge, this is so far the first report of antimicrobial activity of *A. gmelinii* extracts. The phytochemical characteristic of tested extracts was different, but in general only slightly higher activity against Gram-negative bacteria was noticed for ethanolic extract in comparison to the chloroform one. In ethanolic *A. gmelinii* extract the high level of chlorogenic acid was observed. Recent studies showed that chlorogenic acid bound to the outer membrane, disrupted the membrane, exhausted the intracellular potential, and released cytoplasm macromolecules, which led to the death of cell [20]. That may be also an explanation of a more frequent bactericidal activity of ethanolic extract for most of the tested strains. Determining the MBC value allowed to establish the activity to kill bacteria or inhibit its growth. Antimicrobial agents are usually regarded as bactericidal if MBC value is higher no more than four times the MIC value [21]. The low values of MBC/MIC ratio (1–4) for ethanolic extract suggested its bactericidal power for majority of bacterial and fungal strains except for bacteriostatic activity against spore-forming *B. cereus.*

The data shown by Poiata et al. [22] indicated that among ethanol, methanol and hexane extracts from *Artemisia absinthium, Artemisia annua and Artemisia vulgaris* alcoholic extracts were more effective against tested microorganisms. However, all plants’ extracts have moderate or no activity against Gram-negative bacteria. The higher microbial activity of n-hexane, ethyl acetate and menthol extracts observed in other studies, could be related to the higher concentration of active antimicrobial agents like terpenoids, phenolics and volatile oils in *Artermisia* spp. [17, 23].

It is widely thought that some coumarins, including umbelliferone, scopoletin and furanocoumarins play important fungicidal roles in plants. In general, umbelliferone shows no antifungal activities and very weak antibacterial activities irrespective of the test method and sample purity [24–26]. In our study, the chloroform extract of *A. gmelinii* showed higher amount of umbelliferone that may be the cause of lower value of MFCs for tested fungi in comparison to the ethanolic extract.

Standardized MIC testing methods were used by Ahameethunisa and Hopper [10, 17] to determine the antimicrobial activity of *A. nilagirica* and *A. parviflora* extracts from India. Higher activity of both etanolic and chloroform *A. nilagrica* extracts was revealed in their studies in comparison to the one presented in our study what may be caused by different geographical origin of the plants and species and their phytochemical composition. Chloroform extracts of *A. nilagrica* were high in alkaloids, flavonoids, phenols, and terpenoids, similarly to the ethanolic extract which was richer with tannins, saponins and quinines, though [10].

In other studies agar well diffusion method was the only one used to detect antimicrobial activities of *A. annua* leaf extract [19], *A. absinthium* extract [26] and *Artemisia* sp. methanolic extracts recovered by different extraction techniques [18]. The well or disk diffusion methods provide qualitative results, appropriate for screening selection only. This method is not appropriate to give quantitative results allowing for MIC dtermination, because it is not possible to define precisely the amount of the extract diffused into the agar medium. Sometimes it is impossible to distinguish whether the lack of antimicrobial activity is a result of the extract’s action or a lack of its diffusion to agar. Moreover, since the bacterial growth does not mean the bacterial death, this method cannot distinguish bactericidal or bacteriostatic effect of the extracts. We would like to point out that due to the use of standardized methods in antimicrobial activity testing of plant extracts or essential oils, data can be trustworthy and allow researchers to compare the results.

In general, 13 compounds were identified in the analyzed extracts. To the best of our knowledge, this is the first ever work that shows the identification of rhamnazine, umbelliferone, 3-hydroxycoumarin and 4-hydroxycoumarin in the genus *Artemisia*. Moreover, for the first time in *A. gmelinii* species, luteolin, rutin, isorhamnetin and scopolin were identified, even though these substances were previously determined in other species of *Artemisia* [27–29]. The presence of apigenin, scopoletin, chlorogenic acids and two dicaffeoylquinic acid isomers have also previously been reported [12, 13, 30]. Additionally it is worth to highlight that low level of thujone was determined in the tested extract so there should be no potential influence of the activity as well as no toxicity towards the human. Thujone is a natural monoterpene ketone present in variable amounts in a large number of plants, such as *Salvia*, *Tanacetum* and *Artemisia* species. Thujone has neurotoxic action, causing tonic-clonic convulsions and also exhibits hepatotoxicity and porphyrogenic activity [31]. However at the same time it should be noted that the toxic activity of the extracts should not be associated exclusively with thujones as, in addition to the thujone content, the amount and toxicity of other constituents should be taken into consideration when making risk assessment and determining the regulatory status of plants in food and medicines [32].

## Conclusion

There is an urgent need, to work towards the development of safer antimicrobial agents, that are expected to be renewable, non-petrochemical, naturally ecofriendly and easily obtainable. The extracts of wormwood *Artemisia gmelinii* have shown a wide spectrum of antibacterial and antifungal activity. For the first time in *A. gmelinii* species, luteolin, rutin, isorhamnetin and scopolin were identified. Determining of the most potential compounds of *Artemisia gmelinii* can be used to develop effective antibacterial and antifungal agents.

## Supplementary information

**Additional file 1: Figure S1.** HPLC-DAD chromatogram (λ = 325 nm) - chloroform extract from *A. gmelinii* Weber ex Stechm.

**Additional file 2: Figure S2.** HPLC-DAD chromatogram (λ = 325 nm) - ethanolic extract from *A. gmelinii* Weber ex Stechm.

**Additional file 3: Figure S3.** The BPC chromatogram of ethanolic extract of *Artemisia* in negative ionization mode.

**Additional file 4: Figure S4.** The BPC chromatogram of ethanolic extract of *Artemisia* in positive ionization mode.

**Additional file 5: Figure S5.** The BPC chromatogram of chloroform extract of *Artemisia* in negative ionization mode.

## Data Availability

The datasets analyzed during the current study are available from the corresponding author on reasonable request.

## Abbreviations

CID energy: Collision-induced dissociation energy
GACP: Good cultural and collection practices
GC-MS: Gas chromatography mass spectrometry
HPLC-DAD: High performance liquid chromatography with a visible diode array detector
LC-MS: Liquid chromatography-mass spectrometry
MBC: Minimal bactericidal concentration
MFC: Minimal fungicidal concentration
MIC: Minimal inhibitory concentration
