# Supplementary figures and images for: Phytochemical composition of wormwood (Artemisia gmelinii) extracts in respect of their antimicrobial activity
Source: BMC Complement Altern Med. 2019 Oct 28;19:288. doi: 10.1186/s12906-019-2719-x (PMC6819330; doi:10.1186/s12906-019-2719-x)

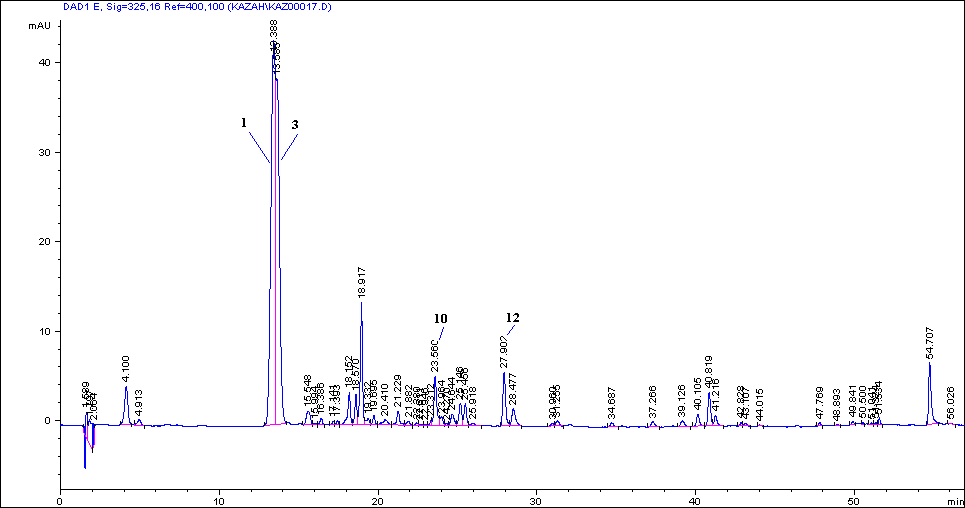


Fig. S1

Supplement: Supplementary file 1 — Additional file 1: Figure S1. HPLC-DAD chromatogram (λ = 325 nm) - chloroform extract from A. gmelinii Weber ex Stechm. [file 12906_2019_2719_MOESM1_ESM.docx]

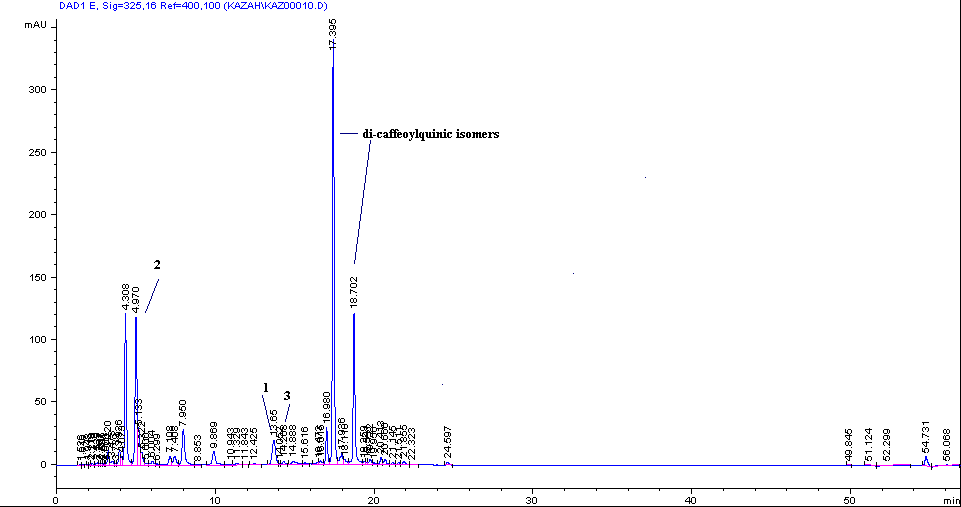


Fig. S2.

Supplement: Supplementary file 2 — Additional file 2: Figure S2. HPLC-DAD chromatogram (λ = 325 nm) - ethanolic extract from A. gmelinii Weber ex Stechm. [file 12906_2019_2719_MOESM2_ESM.docx]
