# Supplementary material for: Phytochemical composition of wormwood (Artemisia gmelinii) extracts in respect of their antimicrobial activity
Source: BMC Complement Altern Med. 2019 Oct 28;19:288. doi: 10.1186/s12906-019-2719-x (PMC6819330; doi:10.1186/s12906-019-2719-x)
Supplement: Supplementary file 4 — Additional file 4: Figure S4. The BPC chromatogram of ethanolic extract of Artemisia in positive ionization mode. [file 12906_2019_2719_MOESM4_ESM.docx]

Fig. S4.
