# Supplementary material for: Phytochemical composition of wormwood (Artemisia gmelinii) extracts in respect of their antimicrobial activity
Source: BMC Complement Altern Med. 2019 Oct 28;19:288. doi: 10.1186/s12906-019-2719-x (PMC6819330; doi:10.1186/s12906-019-2719-x)
Supplement: Supplementary file 5 — Additional file 5: Figure S5. The BPC chromatogram of chloroform extract of Artemisia in negative ionization mode. [file 12906_2019_2719_MOESM5_ESM.docx]

Fig. S5.
